# Recent Advances in Biomass-Based Flame-Retardant Poly(vinyl alcohol) Composites

**DOI:** 10.3390/ma19214594

**Published:** 2026-10-30

**Authors:** Xinyu Chen, Zelin Chen, Zhihao Zhang, Mingli Liu

**Affiliations:** School of Materials Science and Engineering, Beihua University, Jilin 132013, China; 18317819935@163.com (X.C.); chenzelin20210520@163.com (Z.C.); liumingli17@163.com (M.L.)

**Keywords:** poly(vinyl alcohol), bio-based, flame retardant, multifunctional composites, fire safety

## Abstract

Poly(vinyl alcohol) (PVA) is widely used in films, fibers, hydrogels, aerogels, coatings, and adhesives because of its superior transparency, biocompatibility and chemical versatility. Its inherent flammability and potential melt dripping, however, restrict applications that require reliable fire safety. Biomass-derived flame retardants offer a promising route to PVA composites because their hydroxyl, amino and phosphate structures can promote carbonization, release noncombustible gases, scavenge flame-propagating radicals, and reinforce condensed-phase barriers. This review summarizes recent advances in flame-retardant PVA composites based on cellulose, chitosan, lignin, phytic acid, tannic acid, sodium alginate, cyclodextrin, amino acids, and related bio-based components. Their flame-retardant mechanisms, modification strategies, and interactions with inorganic nanomaterials are discussed with emphasis on associated property trade-offs. Current evidence indicates that unmodified biomass components often exhibit insufficient flame-retardant efficiency, limited thermal stability, weak char integrity, and moisture sensitivity when used alone. Phosphorylation, ionic complexation, multiple crosslinking and catalytic carbonization can substantially improve their effectiveness while enabling thermal management, ultraviolet protection, antibacterial activity, hydrophobicity, self-healing, and fire-warning functions. Nevertheless, loss of toughness or transparency, additive leaching and limited long-term validation remain important barriers. Future development should focus on integrated robust interfacial design, durable multifunctionality, scalable processing and application-relevant fire and environmental assessment.

## 1. Introduction

The transition from a fossil-resource-dependent economy toward more sustainable materials has intensified interest in renewable feedstocks, biodegradable polymers, and low-impact manufacturing routes [1]. Within this context, poly(vinyl alcohol) (PVA) is an important water-soluble synthetic polymer that can be integrated with biomass-derived components. Its hydroxyl-rich molecular structure provides excellent film-forming ability, adhesion, transparency, biocompatibility, and chemical reactivity, allowing PVA to be processed into films, fibers, hydrogels, aerogels, coatings, and adhesives [2,3]. These characteristics have supported its use in packaging, textiles, biomedical materials, agriculture, construction, electronic devices, and environmental applications. The growing commercial demand for PVA further increases the need to ensure that PVA-based products satisfy the safety and durability requirements of their intended service environments [4].

Despite these advantages, the inherent flammability of PVA remains a major obstacle to its use in applications that require reliable fire protection [5]. Exposure to heat can induce dehydration, chain scission, and the release of combustible volatile products, followed by ignition and sustained burning. Thermal deformation and melt dripping may further accelerate flame spread or ignite adjacent materials. These hazards are particularly relevant to PVA films, battery separators, electrical and electronic components, thermal management materials, and protective coatings, in which small thicknesses, large exposed surface areas, or contact with heat-generating devices can increase fire risk [6,7]. An effective flame-retardant system must therefore do more than reduce heat release: it should also delay ignition, suppress flame propagation and smoke generation, promote a stable protective char, and maintain the functional properties of the PVA matrix.

Several strategies have been developed to improve the fire performance of PVA, including reactive modification, surface coating, intumescent formulations, inorganic nanofillers, catalytic carbonization, and synergistic multicomponent systems [5]. Conventional halogen-containing flame retardants can provide high efficiency at relatively low cost, but concerns regarding corrosive or toxic combustion products, environmental persistence, and increasingly stringent chemical regulations have restricted their acceptability [8]. Phosphorus–nitrogen intumescent systems are generally more environmentally favorable, yet their high loading, component migration, nonuniform dispersion, and limited compatibility with PVA can impair processability and mechanical performance [9,10]. Inorganic fillers and two-dimensional nanomaterials can reduce heat and mass transfer and reinforce the carbonaceous residue, but efficient protection often requires high filler contents or combination with additional flame retardants. Such formulations may increase strength while markedly decreasing elongation, toughness, transparency, or flexibility [11]. Consequently, the central challenge is not simply to maximize a single fire performance parameter, but to balance flame-retardant efficiency, additive loading, interfacial compatibility, mechanical integrity, optical performance, water resistance, and manufacturability. Bio-based flame retardants provide a promising platform for addressing this challenge because biomass is abundant, chemically diverse, and potentially renewable [12]. Cellulose, chitosan, lignin, phytic acid, tannic acid, sodium alginate, cyclodextrin, starch, and amino-acid derivatives contain hydroxyl, amino, phosphate, carboxyl, or aromatic groups that can contribute to one or more flame-retardant functions. Under fire exposure, these components may promote dehydration and catalytic carbonization, supply carbon or noncombustible gases to an intumescent system, scavenge flame-propagating radicals, or form barriers that restrict the transport of heat, oxygen, and volatile decomposition products. Their abundant functional groups also facilitate hydrogen bonding, ionic complexation, covalent crosslinking, and hybridization with inorganic nanomaterials. These interactions also create opportunities to improve dispersion and char integrity while incorporating additional functions such as antibacterial activity, ultraviolet shielding, thermal or electrical conductivity, self-healing, hydrophobicity, and fire-warning response [13,14].

Existing reviews have addressed flame-retardant PVA materials in general and, separately, the broader development of bio-based flame retardants for different polymer systems. However, a focused review that systematically connects the biomass source and chemical functionality of bio-derived components with their flame-retardant mechanisms, modification strategies, composite architectures, quantitative fire-performance characteristics, and associated property trade-offs in PVA systems remains limited. Accordingly, this review specifically focuses on biomass-based flame-retardant PVA composites and provides a comparative framework covering cellulose, chitosan, lignin, phytic acid, tannic acid, sodium alginate, cyclodextrin, amino acids, and related bio-based components. Particular attention is given to how these materials function as carbon, acid, gas, catalytic, radical-scavenging, or barrier-forming components, and how their performance is altered by phosphorylation, ionic or covalent crosslinking, metal-ion coordination, and hybridization with inorganic nanomaterials. In addition to flame-retardant efficiency, the review critically evaluates associated trade-offs in thermal stability, mechanical properties, transparency, water resistance, processing, and durability, as well as multifunctional features such as thermal management, UV shielding, antibacterial activity, hydrophobicity, self-healing, and fire-warning capability. This framework enables direct comparison of the design strategies, performance advantages, and remaining limitations of biomass-based flame-retardant PVA systems.

The literature reviewed in this work was identified through searches of the Web of Science database, with the final search updated in September 2026. The search focused primarily on publications from 2020 to 2026 in order to capture recent developments in flame-retardant PVA materials. Searches were performed using combinations of “poly(vinyl alcohol)” or “PVA” and “flame retardant” or “flame retardancy”. The retrieved records were subsequently screened manually by examining the titles, abstracts, keywords, and, where necessary, the full texts to identify studies containing at least one bio-based or biomass-derived component. Studies were included if they investigated flame-retardant PVA-based materials containing at least one bio-based or biomass-derived component. Studies focusing exclusively on non-bio-based flame-retardant PVA systems or lacking relevant fire-performance information were excluded. At the same time, the term bio-based” and “biomass-based” flame-retardant PVA systems refer to formulations containing at least one biomass-derived or renewable biological component that makes a meaningful contribution to flame retardancy, char formation, synergistic interactions, interfacial construction, or related multifunctional properties. The system is therefore not necessarily composed exclusively of bio-derived constituents. Formulations containing synthetic flame retardants, metal ions, or inorganic nanomaterials are included when the biomass-derived component plays a functional role in the overall flame-retardant system. Finally, the principal limitations of current formulations are identified, and future priorities are proposed for improving interfacial design, char robustness, durability, multifunctional integration, and application-relevant validation to support the development of safer and more sustainable flame-retardant PVA materials.

## 2. Limitations and Complementarity of Fire-Performance Evaluation Methods

The evaluation of flame-retardant materials commonly involves several standardized or widely used tests, including the limiting oxygen index (LOI), UL-94 classification, cone calorimetry, and related thermal or combustion analyses. Although these methods are useful for characterizing fire-related behavior, they probe different aspects of material response and should not be regarded as interchangeable measures of overall fire safety. In particular, favorable performance in one test does not necessarily imply similarly favorable behavior in another.

The LOI test determines the minimum oxygen concentration required to sustain combustion under specified conditions. It is therefore useful for comparatively assessing the combustibility of materials in an oxygen-controlled environment. However, LOI is measured under relatively simplified laboratory conditions and does not directly represent burning behavior under realistic fire scenarios involving external heat flux, complex ventilation conditions, or dynamic flame spread. Consequently, a high LOI value should primarily be interpreted as an indication of reduced combustibility under the specific test conditions rather than as direct evidence of comprehensive fire safety.

The UL-94 test evaluates the response of a specimen to a relatively small ignition source and is mainly used to assess self-extinguishing behavior, afterflame time, and, depending on the test configuration, dripping characteristics. A UL-94 V-0 classification therefore indicates favorable performance under the prescribed vertical burning conditions. Nevertheless, this classification provides limited information regarding the rate and quantity of heat released during sustained combustion. Thus, achieving a V-0 rating does not necessarily imply low heat-release intensity or low total heat release, and it should not be used alone to demonstrate comprehensive fire safety.

Cone calorimetry provides complementary information by evaluating the heat-release behavior of materials subjected to an imposed external heat flux. Among the commonly reported parameters, the peak heat release rate (pHRR) reflects the maximum intensity of heat release during combustion, whereas the total heat release (THR) represents the cumulative heat released over the course of the test. A lower pHRR generally indicates reduced combustion intensity, while a lower THR suggests a reduction in the overall heat released. However, these parameters are also influenced by the selected external heat flux, specimen configuration, thickness, and testing conditions. In addition, improvements in pHRR or THR do not necessarily correspond to improvements in LOI or UL-94 classification because the underlying test conditions and performance criteria are fundamentally different.

These distinctions are particularly important when comparing different flame-retardant systems. For example, a material may exhibit a high LOI or achieve a UL-94 V-0 rating because of rapid self-extinguishing behavior, while still releasing substantial heat when exposed to sustained external irradiation. Conversely, a system that effectively suppresses pHRR through the formation of a protective char layer may not necessarily exhibit the highest UL-94 classification if ignition or dripping behavior remains unfavorable. Therefore, no single parameter can fully describe the fire-related performance of a material.

Accordingly, the fire-related behavior of flame-retardant materials should be evaluated using multiple complementary metrics. LOI and UL-94 are particularly useful for assessing combustibility and self-extinguishing behavior under defined small-scale conditions, whereas cone calorimetry provides additional insight into heat-release characteristics under external thermal exposure. The interpretation of these results should therefore remain specific to the physical meaning and limitations of each test method, and the term “fire safety” should be used cautiously when conclusions are based only on one or a limited number of laboratory-scale indicators.

## 3. Research Progress in Bio-Based Flame-Retardant PVA

### 3.1. Representative Bio-Based Flame Retardants for PVA

Growing concern for human safety and sustainable development has accelerated restrictions on halogenated flame retardants and intensified development of potentially lower-impact, renewable, efficient, and bio-based alternatives [15,16]. Biomass comprises abundant and diverse natural resources that are sustainable, broadly available, and comparatively inexpensive. Owing to their distinctive compositions and structures, many biomass-derived materials can promote char formation, release noncombustible gases, and scavenge radicals when exposed to flame. Biomass-derived flame retardants therefore represent promising environmentally compatible candidates for fire-safety applications. Representative biomass materials investigated in flame-retardant PVA composites include cellulose, chitosan, lignin, phytic acid, sodium alginate, and tannic acid. The following sections review their contributions to the flame retardancy of PVA and discuss the limitations of current bio-based systems.

(1) Cellulose. Cellulose is the principal structural component of the cell walls of plants, bacteria, fungi, and algae and is the most abundant natural biopolymer, with an estimated annual production of approximately 1.110–1.111 tonnes [17]. It consists of linked glucose units, with a degree of polymerization ranging from 100 to 40,000 depending on botanical source and extraction method. Its hydroxyl-rich structure can generate carbonaceous residue under appropriate thermal conditions, making cellulose a potential carbon source in flame-retardant systems [18]. Hasan et al. fabricated microcrystalline-cellulose/PVA (MCC-PVA) films by solution casting [19]. A film containing 80% MCC and 20% PVA produced only 3 wt% char at 700 °C and failed the vertical-burning test, demonstrating the limited performance of unmodified cellulose. Phosphorylation markedly improves its activity. Pan et al. reacted cellulose with phosphoric acid in molten urea to prepare a biodegradable flame retardant (PCA) [20]. At 15 wt% loading, PVA15 reached an LOI of 30%, achieved UL-94 V-0, and yielded 19.71 wt% char at 700 °C. Wei et al. similarly prepared phosphorylated cellulose (PCF), crosslinked it with glutaraldehyde, and freeze-dried the formulation into an aerogel [21]. PVA/PCF10 achieved UL-94 V-0, an LOI of 30%, and a 2.3-fold increase in char yield at 700 °C. Zhang et al. introduced bifunctional carbon dots (CDFP) into PVA/oxidized nanofibrillated cellulose (ONFC) films [22]. At a fixed PVA:ONFC mass ratio of 100:1, synergy between ONFC and CDFP enabled PVA-ONFC@CDFP10 to reach an LOI of 33.4% and a UL-94 VTM-0 rating. In such systems, the phosphorus functionality mainly promotes catalytic dehydration and carbonization, whereas crosslinking can help stabilize the polymer network and improve the continuity and mechanical integrity of the resulting char. The observed improvement in fire performance therefore reflects a coupled chemical and structural effect rather than phosphorus content alone.
(2) Chitosan. Chitosan (CH) is a fully or partially deacetylated derivative of chitin, the second most abundant natural polysaccharide. Its amino and hydroxyl groups allow it to act as both a gas and carbon source. At elevated temperature, amino groups release noncombustible gases such as NH_3_ and N_2_, while hydroxyl-containing chains undergo dehydration, carbonization, and ring-opening reactions that generate aromatic structures and a compact condensed-phase char. Because CH alone has limited efficiency, it is commonly combined with other components in synergistic or intumescent systems. Liu et al. prepared a gel from water-soluble chitosan (WSC), poly(vinyl alcohol)-based macromonomer (PVAM), acrylic acid (AA), and Cu^2+^ and used it as a flame-retardant coating for polyester fabric [23]. The pHRR decreased from 329.5 W/g for the uncoated fabric to 197.2 W/g with WSC/PVAM/AA and to 138.9 W/g with WSC/PVAM/AA/Cu^2+^, indicating synergy between WSC and Cu^2+^. Tan et al. modified PVA with a hexachlorocyclotriphosphazene derivative (SHCP) and chitosan (CS), then crosslinked the composite with Cu^2+^ to obtain PVA/SHCP@CS-Cu [24]. PVA/SHCP@CS and PVA/SHCP@CS-Cu reduced pHRR from 247.40 to 187.48 and 117.82 kW/m^2^, respectively; the reported THR values changed from 8.46 to 8.26 and 7.11 MJ/m^2^, respectively. Ning et al. prepared HTPA through electrostatic and hydrogen-bonding interactions between PA and a chitosan derivative (HTCC) [25]. At 12 wt% HTPA, the LOI of PVA-HTPA12 increased from 18.27% to 33.30%, exceeding the values of PVA-HTCC12 (20.30%) and PVA-PA12 (31.33%) and supporting synergy between PA and HTCC.
(3) Lignin. Lignin is one of the three principal constituents of wood and one of the most abundant biopolymers after cellulose and chitin [26]. Its complex structure contains methoxy, carbonyl, and aromatic groups and allows it to serve as a carbon source in intumescent systems. During combustion, methoxy-group decomposition can generate radical-scavenging species and noncombustible gases, while aromatic and carbonyl structures contribute to a stable protective char [27]. Lignin can therefore act in both the gas and condensed phases. Ingtipi et al. prepared a NaOH/borax-crosslinked hydrogel (PBXNL) from PVA, xanthan gum (XG), and lignin nanoparticles (LNPs) [28]. Increasing the LNP content increased char production and progressively reduced pHRR and THR. Shu et al. modified APP with lignin to produce L-APP and melt-blended it with thermoplastic PVA (TPVA) [29]. At 20 wt%, both APP and L-APP enabled UL-94 V-0, but PVA/L-APP20% produced more char and showed better mechanical performance, suggesting that lignin improved both APP-TPVA compatibility and carbonization.
(4) Phytic acid. Phytic acid (PA), one of the most frequently investigated biomass-derived flame retardants for PVA, occurs naturally in plant seeds, grains, and legumes. Its six phosphate groups provide a phosphorus content of up to 28% [30]. During combustion, PA generates phosphorus-containing radicals that scavenge OH· radicals in the gas phase. Its phosphorus-containing decomposition products also catalyze polymer dehydration and carbonization and remain in the residue, improving char stability. Lao et al. solution-cast PVA/okra-polysaccharide (OP)/PA films (POP) [31]. Radical scavenging and catalytic carbonization by PA enabled POP to achieve UL-94 V-0 and an LOI of 42.5%. Zhao et al. directly incorporated PA into PVA as a flame-retardant coating for wood [32]. PA promoted gelation and improved the mechanical and fire performance of the wet coating, although dried PVA-15PA showed a marked increase in pHRR. This limitation motivates combination of PA with nitrogen-containing compounds. Wang et al. reacted PA with creatine (CR) to prepare an integrated bio-based phosphorus–nitrogen flame retardant (PACR) and solution-cast PVA/PACR films [33]. At 20 wt% PACR, PVA/PACR-20 achieved UL-94 V-0, its LOI increased from 18.4% to 29.5%, and its pHRR and THR decreased by 92% and 68%, respectively.
(5) Tannic acid. Tannic acid (TA) is an abundant plant-derived polyphenol. Its high carbon content and multiple phenolic hydroxyl groups make it a useful bio-based char former. During combustion, TA carbonizes into a comparatively stable residue, while phenoxy radicals can scavenge reactive radical species and terminate combustion reactions [34,35]. TA therefore contributes to both the condensed and gas phases. Lin et al. compression-molded blends of PVA, TA, and resveratrol (RE) (RETA-PVA) [36]. Char yield increased with TA content, although the blends did not pass the vertical-burning test, showing that TA generally requires hybridization or combination with other components. Abdelkhalik et al. synthesized a melamine phosphate salt of tannic acid (MTP) and incorporated it into PVA [37]. At 15 wt% MTP, the LOI increased from 18.8% to 39.9% and the film achieved UL-94 V-0. De la Cruz et al. introduced sodium alginate (SA) and TA into PVA and then crosslinked the network with CaCl_2_ and boric acid to prepare PVA/SA/TA aerogels [38]. TA promoted char formation through ester-bond cleavage, release of benzenediol/triol species, and oxidation of phenolic substrates. The resulting char yield reached 61.3 wt%, and pHRR decreased by 74.5%.
(6) Sodium alginate. Sodium alginate (SA) is an intrinsically flame-resistant biopolymer that promotes catalytic carbonization and suppresses the release of combustible gases [39]. Zhang et al. freeze-dried cellulose (Cel), PVA, and SA to prepare Cel-PVA-SA composite aerogels. In contrast to Cel-PVA, the SA-containing aerogel self-extinguished during vertical burning [40]. Guo et al. combined magnesium hydroxide (MH) and SA to flame-retard agar (AG)/PVA aerogels (AP-MS) [41]. The MH/SA combination improved mechanical and fire performance, produced a more continuous and compact residue than MH alone, and enabled a UL-94 V-0 rating.
(7) Other bio-based flame retardants. β-Cyclodextrin, starch, and amino acids provide additional renewable options. Among α-, β-, and γ-cyclodextrin, β-cyclodextrin (β-CD) is comparatively inexpensive. Its hydroxyl-rich structure dehydrates and carbonizes during combustion, producing a protective residue that restricts oxygen and heat transfer. Xing et al. wet-spun PVA/β-CD fibers and crosslinked them with hexamethylene diisocyanate (HDI) to obtain PVA/CD/HDI fibers [42]. The fibers reached an LOI of 41.7%, reduced pHRR by 77.7%, and showed smoke-suppression capability, which was attributed to the dense char generated by the crosslinked network. Several amino acids, including serine, aspartic acid, arginine, and tyrosine, have also demonstrated flame-retardant activity [43,44,45,46]. Their nitrogen-containing structures release noncombustible gases that dilute oxygen and flammable volatiles. Liu et al. prepared PVA/L-aspartic-acid phosphate ionic-liquid (AAIL) films (PL) by a sol–gel route [47]. At 20 wt% AAIL, PL-20 achieved UL-94 V-0 and an LOI of 30.1%.

To provide a clearer cross-comparison of the biomass-derived flame-retardant systems reviewed in this section, their principal formulation characteristics and representative flame-retardant performance parameters are summarized in Table 1. The compiled data include biomass source, chemical modification, additive loading, composite type, LOI, UL-94 classification, pHRR, and char yield, where available. The comparison reveals considerable variation in the reported performance among different systems, which can be attributed to differences in chemical composition, flame-retardant loading, composite architecture, and the dominant flame-retardant mechanism. It should also be emphasized that LOI, UL-94 classification, pHRR, and char yield describe different aspects of combustion behavior. Consequently, a formulation exhibiting the highest value in one parameter should not necessarily be regarded as the overall best-performing system.

**Figure 1:**
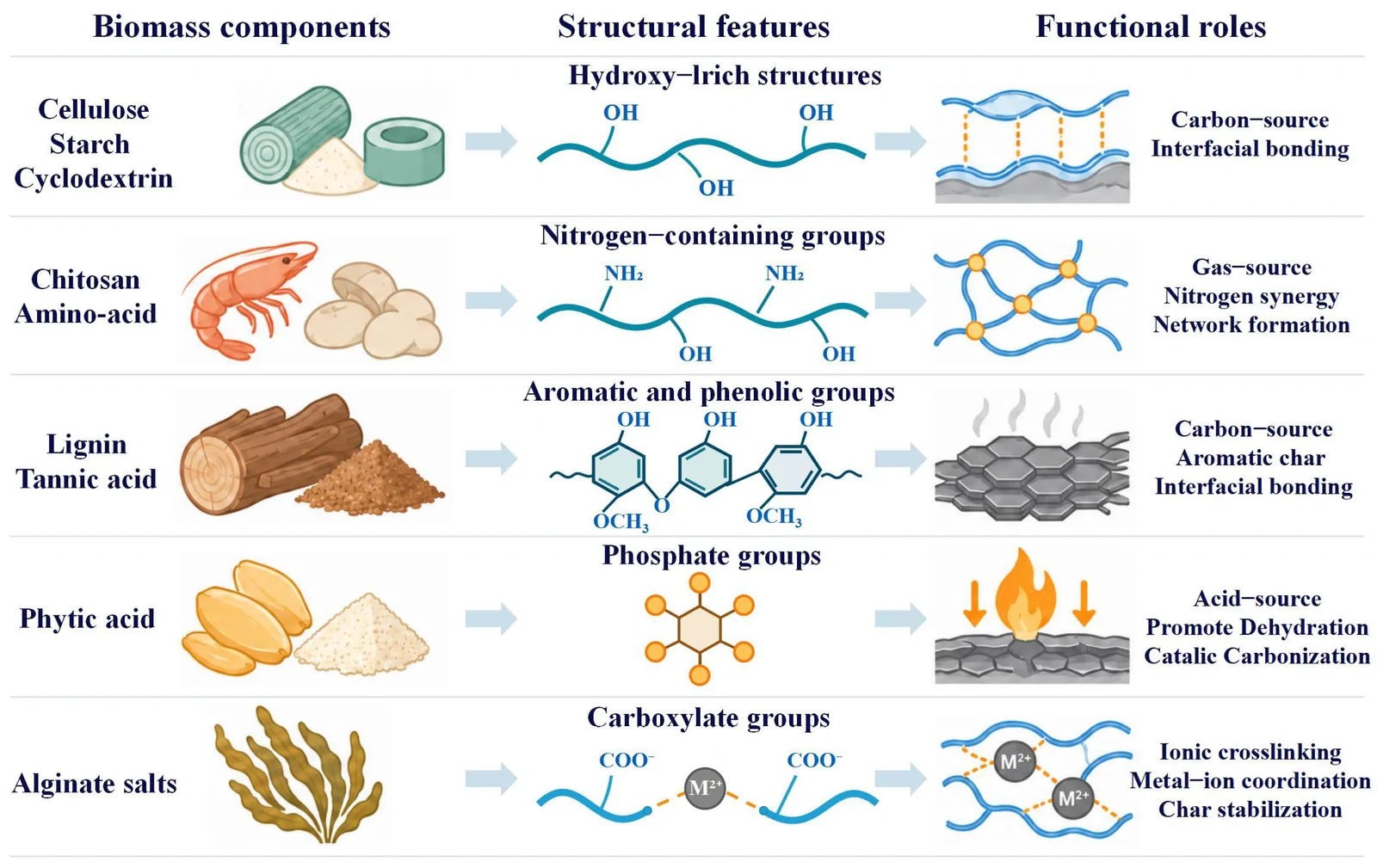
The structure features and functional roles of different biomass. (ChatGPT (OpenAI, GPT6.0) was used to assist in generating the preliminary graphical elements, visual design, and layout of this schematic illustration. The AI-assisted figure was subsequently reviewed and edited by the authors to ensure scientific accuracy).

From a structure-function perspective, the biomass-derived components discussed above can be broadly divided according to their dominant flame-retardant roles. As shown in Figure 1, Hydroxyl-rich polysaccharides such as cellulose and cyclodextrin primarily act as carbon sources and require catalytic activation or crosslinking to generate an effective protective char. Aromatic or polyphenolic components, including lignin and tannic acid, possess greater intrinsic char-forming ability because their aromatic structures favor thermally stable carbonaceous residues, while radical-scavenging species may additionally contribute in the gas phase. Nitrogen-containing biopolymers such as chitosan and amino-acid derivatives provide both condensed-phase carbonization and gas-phase dilution through the release of noncombustible gases. Phytic acid is intrinsically active because its high phosphate-group density enables both catalytic dehydration/carbonization and gas-phase radical scavenging, whereas sodium alginate mainly promotes condensed-phase catalytic carbonization. Accordingly, flame-retardant efficiency is governed less by the biomass fraction itself than by the density and spatial organization of phosphorus-, nitrogen-, aromatic-, hydroxyl-, and carboxyl-containing groups, as well as by crosslinking and interfacial interactions that determine char continuity and stability. The effective loading is therefore strongly system-dependent. Representative high-performance formulations range from 12 wt% HTPA (LOI 33.3%) and 15 wt% phosphorylated cellulose or MTP (UL-94 V-0) to approximately 20 wt% for several phosphorus–nitrogen systems, while synergistic formulations such as PVA/APP10/SA5 demonstrate that a biomass-derived synergist can be effective at substantially lower loading. Thus, chemical functionality and cooperative condensed-/gas-phase mechanisms are more informative design parameters than additive concentration alone.

**Figure 2:**
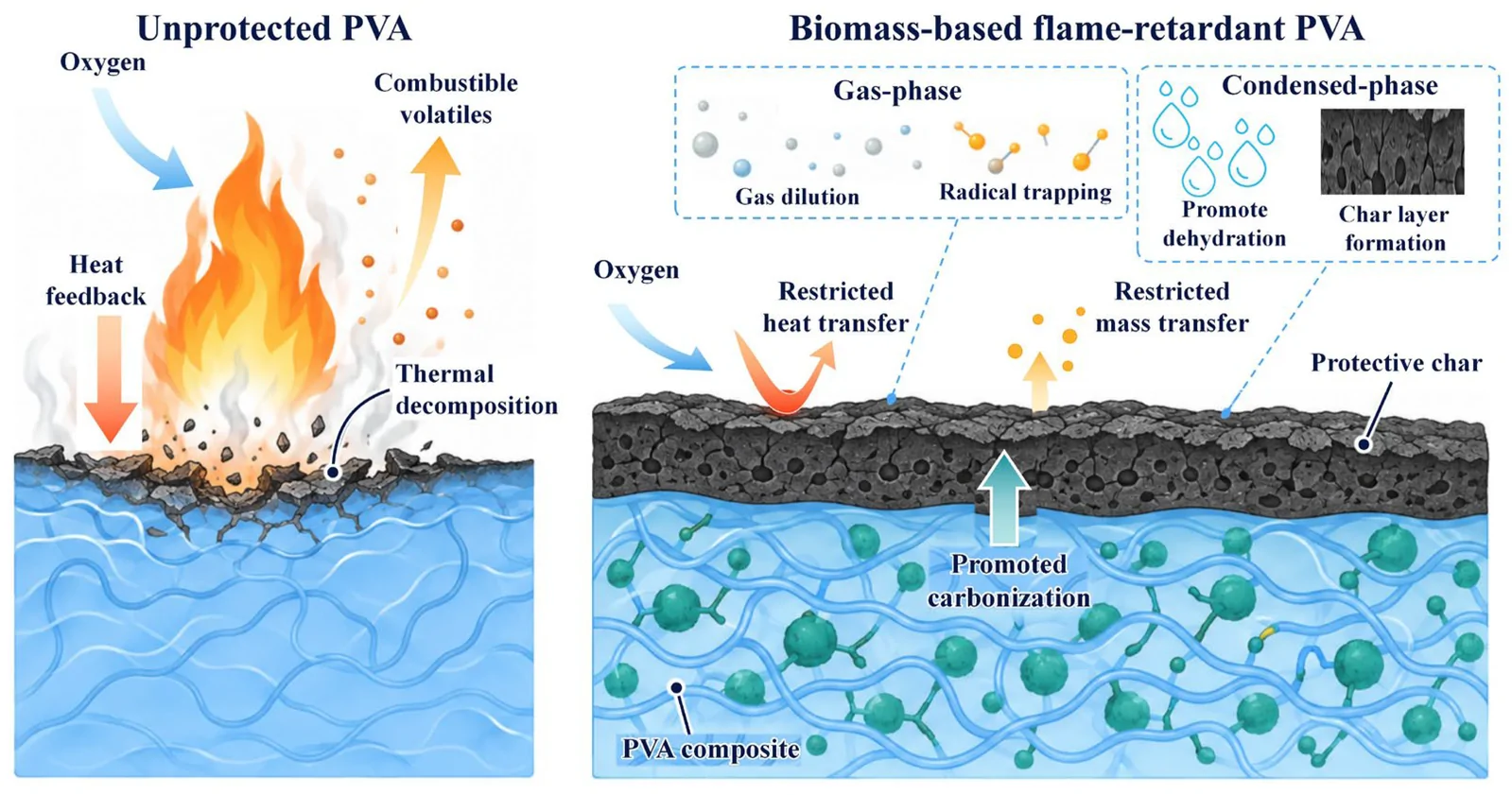
The potential flame-retardant mechanism of biomass-based PVA composites. (ChatGPT (OpenAI, GPT6.0) was used to assist in generating the preliminary graphical elements, visual design, and layout of this schematic illustration. The AI-assisted figure was subsequently reviewed and edited by the authors to ensure scientific accuracy).

As summarized in Figure 2, biomass-based flame-retardant PVA composites interrupt the combustion cycle through condensed-phase protection and, in suitable formulations, complementary gas-phase mechanisms. Upon heating, unprotected PVA undergoes thermal decomposition and releases combustible volatiles, while heat feedback from the flame sustains further degradation. Incorporating appropriately designed biomass-derived components can redirect decomposition toward dehydration and carbonization. Phosphorus-containing acidic species promote char formation from PVA and hydroxyl-rich biomass, whereas aromatic components such as lignin and tannic acid contribute to the carbonaceous residue. Interfacial interactions and crosslinked networks can further support the development of a coherent protective layer. This layer reduces heat transfer to the underlying matrix and restricts oxygen ingress and the outward transport of combustible decomposition products, thereby weakening the feedback between pyrolysis and flaming combustion. In some formulations, noncombustible gases dilute the combustible gaseous mixture, while suitable phosphorus-containing decomposition species can interfere with flame-propagating radical reactions. These gas-phase contributions depend on the chemical composition and decomposition pathway and should not be assumed for every biomass-based system. Consequently, effective protection requires more than an increased char yield: the residue must also maintain sufficient continuity and stability to sustain its barrier function during fire exposure.

Comparison among the reported strategies suggests that unmodified biomass components generally provide limited flame-retardant efficiency when used alone, whereas chemical modification and multicomponent synergistic systems achieve more substantial improvements. Phosphorylation and phosphorus-containing systems are particularly effective in promoting dehydration and catalytic carbonization, while phosphorus–nitrogen combinations further integrate condensed-phase charring with gas-phase dilution. Ionic or covalent crosslinking, metal-ion coordination, and bio-based/inorganic hybridization can additionally enhance char integrity and barrier performance, although these approaches usually increase formulation and processing complexity.

The literature demonstrates substantial progress toward greener biomass-based flame-retardant PVA composites, but three limitations remain. First, many bio-based additives have modest flame-retardant efficiency and thermal stability when used alone and must be combined with phosphorus-containing or nitrogen-containing components in intumescent systems. Although this improves fire performance, separation of the acid, carbon, and gas sources complicates processing and increases cost, and some auxiliary phosphorus- or nitrogen-containing additives are not environmentally benign. Second, chars derived from unmodified biomass can be loose and unstable, limiting their ability to block heat and combustible volatiles. Third, poor water resistance can cause leaching and loss of flame-retardant performance.

### 3.2. Multifunctional Bio-Based Flame Retardants for PVA

To address these limitations, researchers have hybridized multiple biomass-derived materials or combined biomass components with inorganic materials before incorporation into PVA. Such strategies aim to overcome the weaknesses of individual bio-based and inorganic flame retardants, improve flame-retardant efficiency, and introduce additional functions, including thermal and electrical conductivity, antibacterial activity, self-healing, and electromagnetic shielding. Fully bio-based systems and bio-based/inorganic hybrid flame retardants are therefore receiving increasing research attention.

Thermally conductive PVA composites are particularly relevant to electrical, electronic, and wearable devices, where heat dissipation affects service life and operational reliability. Wang et al. synthesized h-BN@PDA@TiO_2_ hybrid nanoparticles from hexagonal boron nitride (h-BN), polydopamine (PDA), and TiO_2_ and used them to improve the thermal conductivity and fire performance of PVA [48]. The high intrinsic conductivity of h-BN and the compact char promoted by h-BN@PDA@TiO_2_ increased thermal conductivity by 239.1% and reduced pHRR by approximately 25.5%. Chen et al. covalently grafted sucrose onto boron nitride nanosheets (BNNSs) to produce a filler (sucrose-g-BNNSs) with uniform dispersibility in water and organic solvents [49]. Its incorporation improved both heat dissipation and flame retardancy. Wang et al. used lignin nanoparticles (LNPs) to enhance BN-OH/PVA films [50]. Adding 7.1 vol% LNPs increased the onset decomposition temperature from approximately 260 to 310 °C and enabled self-extinguishing behavior. A smaller addition of 2.5 vol% LNPs filled interfacial voids, reduced trapped air, and increased the thermal conductivity of the composite film.

Packaging films and adhesives may additionally require transparency, resistance to mold, antibacterial activity, and ultraviolet (UV) protection. Ma et al. prepared a high-performance wood adhesive (SM/PVA/HT) from soybean meal (SM), PVA, and hydroxyapatite (HA)/tannic acid (TA) hybrid nanoparticles (HT) [51]. Synergy between the inorganic and phenolic components enabled SM/PVA/HT-10% to self-extinguish and remain free of visible mold after 15 days under ambient conditions. Inhibition-zone diameters against Escherichia coli and Staphylococcus aureus were 9.0 and 16.7 mm, respectively. Liu et al. used SA as a synergist for APP-containing PVA [52]. PVA/APP10/SA5 outperformed PVA/APP0/SA15 and PVA/APP15/SA0 in fire tests and self-extinguished during vertical burning. Coatings on leaves and foams largely retained transparency, while UV-visible spectroscopy confirmed both high visible transparency and UV absorption. A smartphone fitted with the film remained responsive to touch input, suggesting potential for protective packaging and coatings in electronic devices. Zhao et al. crosslinked amino-functionalized halloysite nanotubes (A-HNTs), CS, and PA within PVA to form an organic–inorganic PA/A-HNT/CS/PVA film [53]. Relative to neat PVA, tensile strength increased from 19.8 to 31.0 MPa, char yield at 700 °C reached 26 wt%, LOI increased from 18.5% to 32.2%, and the film achieved UL-94 V-0. PA, CS, and the dual-crosslinked network also reduced UV-region transmittance from 90% to below 15%.

Water resistance is an important consideration for biomass-based flame-retardant PVA materials because both PVA and many biomass-derived additives are intrinsically hydrophilic, while physically incorporated inorganic flame retardants may be susceptible to migration or leaching from the matrix. Wang et al. prepared a chemically and physically double-crosslinked PVA/PA/MMT aerogel [54], in which the PA/MMT network contributed to flame retardancy and thermal insulation. Subsequent chemical vapor deposition of a methyltrichlorosilane layer increased the water contact angle from 76° to 130°, demonstrating a substantial improvement in surface hydrophobicity. Yang et al. freeze-dried CS, alumina, and PVA to prepare CAP aerogels and investigated the effect of alumina content [55]. The CAP aerogel exhibited improved flame retardancy compared with CP, with an LOI of 41.1%, although the hydroxyl-rich CA1.5P surface remained highly hydrophilic. After methyltrimethoxysilane treatment, the water contact angle increased from 0° to 134° and remained at 128° after 24 h at a relative humidity above 50%, indicating persistent surface hydrophobicity under the reported humidity condition. Although the siloxane modification caused an approximately 5% decrease in LOI, the hydrophobic aerogel retained a UL-94 V-0 rating. The enhanced surface hydrophobicity may help reduce moisture uptake and suppress moisture-induced degradation or additive leaching, thereby contributing to improved durability under humid conditions. Further fire testing after prolonged humidity exposure, water immersion, or repeated wet–dry cycles is still needed to establish long-term flame-retardant durability.

Self-healing can preserve the performance of flame-retardant PVA coatings after cracking. Zhao et al. prepared a PVA/PA/MXene hydrogel by freeze–thaw cycling and coated it onto wood [56]. Relative to bare wood, the coated substrate achieved UL-94 V-0, its time to ignition increased from 32 to 69 s, and its pHRR and THR decreased by 41.6% and 36.14%, respectively. MXene increased the density of dynamic, reversible hydrogen bonds. After cutting, contact with water enabled these bonds to reform across the interface and restored coating continuity. Zhou et al. prepared a borax-crosslinked gel (PBSL) from SA, alkali lignin (AL), and PVA for suppressing spontaneous combustion in coal mines [57]. Complete encapsulation isolated coal from oxygen and reduced its temperature from approximately 850 to 237 °C within 10 min. The gel lost only 58 wt% of its mass at 800 °C. Dynamic borate bonds also re-formed when freshly cut surfaces were brought into contact, enabling self-healing.

Fire-warning capability provides an additional layer of protection and generally requires electrical conductivity. Liu et al. prepared a flame-retardant hydrogel from calcium alginate (CA), PVA, and expandable graphite (EG) [58]. Water evaporation removed heat, while EG and CA promoted an expanded, compact char that restricted heat transfer. The pHRR decreased from 264.9 to 67.2 W/g and THR from 15.4 to 12.1 kJ/g. Expansion of EG during burning created a conductive pathway that illuminated an LED and generated a warning signal. Xia et al. modified carbon black (CB) with vanillin and phosphate by in situ polymerization to form core–shell CB@KF nanoparticles, then combined them with PVA and carbon nanotubes (CNTs) as a cotton-fabric coating and flexible fire-warning sensor (CB@KF-CF) [59]. After vertical burning and cone calorimetry, the char length was 7.8 cm and THR was reduced by more than 50%. Response times to an abnormal temperature of 350 °C and to an alcohol flame were 8 and 4 s, respectively.

**Figure 3:**
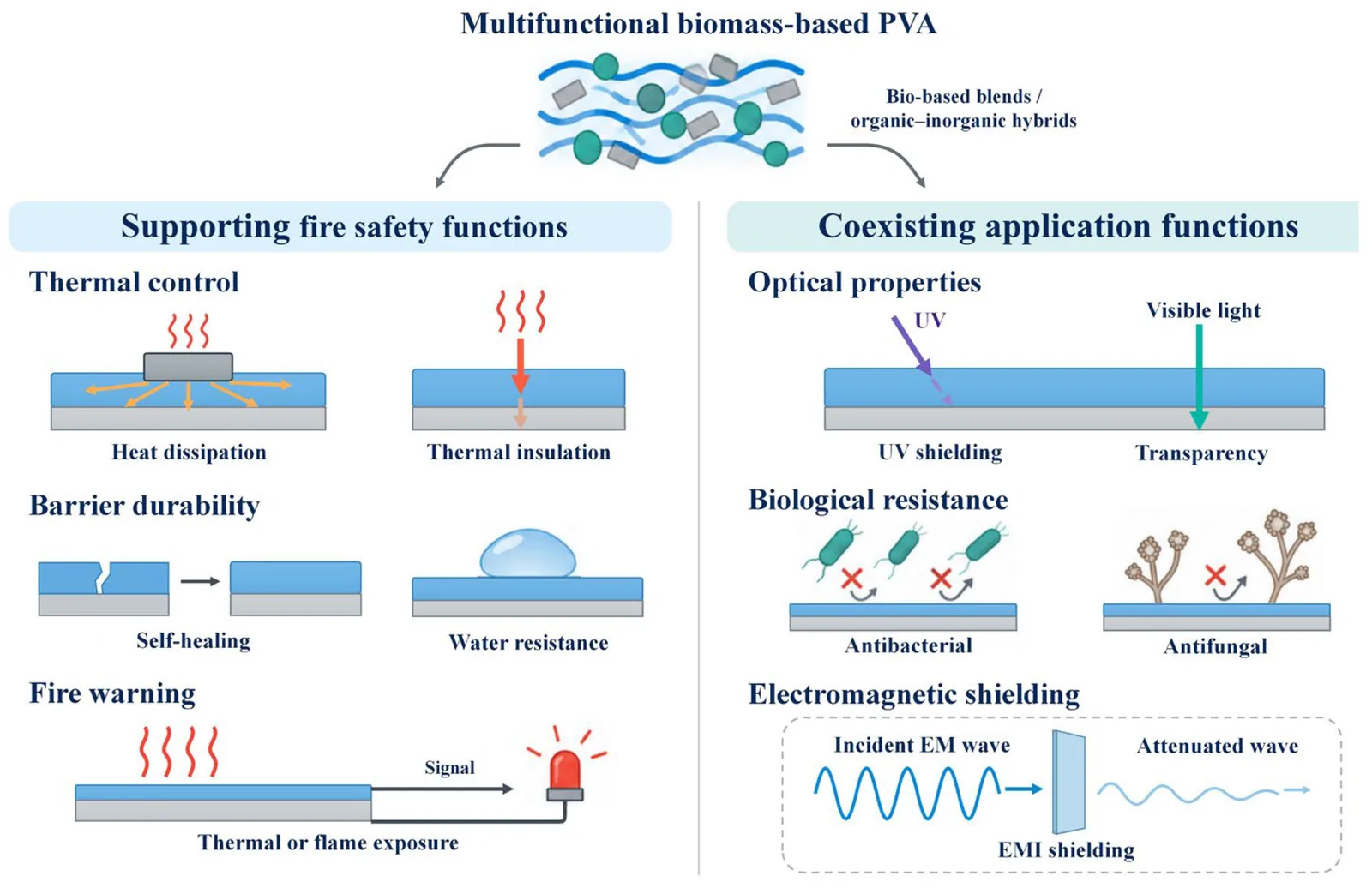
Multifunctional design of biomass-based flame-retardant PVA composites and its relationship with flame retardancy. (ChatGPT (OpenAI, GPT6.0) was used to assist in generating the preliminary graphical elements, visual design, and layout of this schematic illustration. The AI-assisted figure was subsequently reviewed and edited by the authors to ensure scientific accuracy).

In summary, as shown in Figure 3, the additional functions reported for biomass-based flame-retardant PVA systems can be divided into two categories according to their relationship with fire safety. Some functions directly or indirectly contribute to fire protection. For example, thermal management can reduce local heat accumulation, thermal insulation can retard heat transfer, fire-warning behavior can provide an early indication of thermal or flame exposure, and self-healing can help restore the integrity of protective coatings after damage. Hydrophobicity may also improve the long-term durability of flame-retardant systems by reducing moisture uptake and additive leaching. In contrast, properties such as antibacterial activity, UV shielding, transparency, and electromagnetic shielding generally do not directly enhance flame retardancy; rather, they coexist with fire protection and increase the functional value and application versatility of the material. Thus, these additional functions should be distinguished between those that support fire protection and those that primarily broaden the application scope of the composite.

## 4. Conclusions

Biomass-derived components provide diverse routes for improving the fire performance of PVA through condensed-phase carbonization, gas-phase dilution or radical trapping, catalytic effects, and physical barrier formation. Cellulose and cyclodextrin mainly function as hydroxyl-rich carbon sources; chitosan and amino-acid derivatives can provide carbon and nitrogen-containing species; lignin and tannic acid favor aromatic char formation; phytic acid serves as an effective phosphorus-containing acid source; and sodium alginate contributes to catalytic carbonization and structural stabilization. Their abundant functional groups also enable hydrogen bonding, ionic complexation, covalent crosslinking, and hybridization with inorganic phases.

Comparison of the reported systems indicates that single, unmodified biomass components rarely provide an optimal balance between flame retardancy and material performance. More effective approaches generally involve phosphorylation, phosphorus–nitrogen synergy, ionic or covalent crosslinking, metal-ion coordination, or bio-based/inorganic hybridization, which improve catalytic carbonization, char integrity, and heat- and mass-transfer barriers. These strategies can substantially improve LOI, vertical-burning behavior, heat-release characteristics, smoke suppression, and char formation while also enabling additional functionality.

Nevertheless, significant trade-offs remain. Increased flame-retardant loading or formulation complexity can impair flexibility, toughness, transparency, processability, and recyclability, while the hydrophilicity of PVA and many biomass-derived additives can compromise durability through moisture uptake and additive leaching. Furthermore, differences in formulation, specimen geometry, conditioning, and fire-testing methods limit direct quantitative comparison among published systems. Overall, the key challenge is to achieve effective and durable fire protection while retaining the intrinsic functionality and processability of PVA.

## 5. Future Perspectives

(1) High efficiency at low additive loading. A primary objective is to reduce the quantity of flame retardant required to achieve reliable protection. High loadings increase cost, complicate processing, and often diminish the intrinsic flexibility, transparency, and film-forming ability of PVA. Future formulations should integrate acid, carbon, gas, catalytic, and barrier functions within a single-molecule or supramolecular architecture. Biomass-derived polyelectrolyte complexes, covalently modified biopolymers, and self-assembled organic–inorganic hybrids may reduce component separation and improve the spatial coordination of these functions. Composition–structure–property relationships should be established to determine whether performance gains arise from true synergy rather than from the additive contributions of individual components.
(2) Smoke production and combustion toxicity also require greater attention. Improved flame retardancy does not necessarily imply reduced smoke generation, and lower heat release cannot be directly interpreted as lower smoke toxicity. At present, systematic evaluation of combustion-smoke toxicity in biomass-based flame-retardant PVA systems remains limited, particularly with respect to in vivo toxicological assessment. Future studies should therefore combine conventional fire-performance tests with smoke-density measurements, gas-phase analysis, and appropriate toxicity evaluation to obtain a more comprehensive assessment of fire safety.
(3) Interfacial engineering and robust char formation. The quality of the condensed-phase residue is central to the performance of biomass-based systems. A loose, cracked, or discontinuous char cannot effectively restrict heat, oxygen, and volatile-fuel transport. Interfacial interactions among PVA, biomass-derived components, metal ions, and inorganic nanofillers should therefore be controlled through hydrogen bonding, ionic coordination, covalent crosslinking, and compatible surface functionalization. Particular attention should be given to the continuity, expansion behavior, mechanical strength, oxidation resistance, and thermal stability of the char. Combining real-time thermal analysis with chemical and morphological characterization of residues would help link decomposition pathways to barrier development and identify the processes that govern flame extinction.
(4) Water resistance and long-term durability. The hydrophilicity of PVA and many biomass-derived flame retardants remains a major obstacle to practical use. Moisture absorption can plasticize the matrix, weaken interfaces, promote additive migration or leaching, and change both mechanical and fire performance. Hydrophobic modification, double-network crosslinking, encapsulation, and durable surface barriers provide possible solutions, but they should not compromise biodegradability, recyclability, transparency, or adhesion. Future studies should evaluate performance after water immersion, humidity conditioning, thermal cycling, ultraviolet exposure, abrasion, repeated deformation, and realistic service aging. Repeated fire-warning or self-healing cycles should also be examined when these functions are claimed.
(5) Application-oriented multifunctional integration. Multifunctionality should be designed around a defined end use rather than added as an isolated laboratory feature. Electronic and wearable devices require flame retardancy together with thermal conductivity, electrical safety, flexibility, and stable sensing behavior. Packaging and coating applications may additionally require transparency, ultraviolet protection, antimicrobial activity, adhesion, low migration, and resistance to moisture. Construction and fire-protection materials require dimensional stability, thermal insulation, weathering resistance, and reliable performance at larger thicknesses and areas. Future work should identify the minimum set of mutually compatible functions needed for each application and evaluate inevitable trade-offs among fire protection, mechanical performance, optical behavior, transport properties, and environmental durability.
(6) Scalable processing and comprehensive sustainability assessment. Most reported systems are prepared by laboratory-scale solution casting, freeze-drying, repeated assembly, or multistep chemical modification. Translation to industrial production will require shorter processing routes, lower water and energy consumption, inexpensive feedstocks, reproducible raw-material quality, and compatibility with continuous coating, extrusion, fiber spinning, or film manufacture. Fire performance should be evaluated using consistent conditioning procedures and complementary tests, including limiting oxygen index, vertical burning, calorimetry, smoke production, and toxicity analysis, because no single metric captures overall fire safety. Life-cycle assessment, biodegradation behavior, recyclability, potential release of nanofillers or metal ions, and end-of-life scenarios should be considered together with flame-retardant efficiency. Such integrated evaluation is essential for determining whether a nominally bio-based formulation provides a genuine environmental advantage over conventional alternatives.

## Figures and Tables

**Table 1:** Summary of biomass-derived flame-retardant systems and their flame-retardant performance in PVA composites.

Biomass Source	Chemical Modification	Loading (wt%)	Composite Type	LOI (%)	UL-94	pHRR	Char Yield at Temperature and Atmosphere (wt%, °C)	Ref.
Cellulose	MCC	80	Film	NR	NR	NR	3.0, 700, N_2_	[19]
	PCA	15	Film	30.0	V-0	NR	19.7, 700, Air	[20]
Chitosan	PVAM/AA	30	Coating	27.2	V-0	NR	28.4, 600, Ar	[23]
	HTCC/PA	12	Film	33.3	V-0	NR	19.5, 600, N_2_	[25]
Lignin	XG/LNPs	23	Hydrogels	39.0	V-0	90.3 kW/m^2^	42.0, 600, N_2_	[28]
Phytic acid	OP	43	Film	42.5	V-0	NR	30.0, 600, N_2_	[31]
	PA, CR	20	Film	29.5	V-0	42.0 W/g	30.0, 600, N_2_	[33]
Tannic	MTP	15	Film	39.9	V-0	NR	14.1, 700, N_2_	[37]
	TA/SA	50	Aerogels	NR	V-0	112.5 kW/m^2^	61.3, 600, N_2_	[38]
Sodium alginate	PNIPAAm/SA	67	Hydrogels	29	V-0	NR	36.0, 600, N_2_	[39]
	MH/SA	50	Aerogels	34.1	V-0	91.1 kW/m^2^	42.9, 600, N_2_	[41]

NR: No Report.

## Data Availability

No new data were created or analyzed in this study. Data sharing is not applicable to this article.
